# Steady-State Fluorescence and Excitation–Emission Matrix Analysis of Sunlight-Assisted Silver Nanoparticle Formation Using *Cucurbita pepo* Peel Extract

**DOI:** 10.3390/nano16211387

**Published:** 2026-10-29

**Authors:** Fatehia S. Alhakami, Ashjan M. Alharbi, Aliaa Alrashidi, Mohammed S. Alotaibi, Ehssan Moglad, Nourah A. Albarrak, Afnan A. Alamri, Emre Cevik, Amani M. Alansi, Talal F. Qahtan

**Affiliations:** 1 Department of Physics, College of Science and Humanities in Al-Kharj, Prince Sattam Bin Abdul Aziz University, Al-Kharj 11942, Saudi Arabia; f.alhakami@psau.edu.sa (F.S.A.); m.alotiaby@psau.edu.sa (M.S.A.); 2 Department of Physics, College of Science, Qassim University, Buraydah, Saudi Arabia; aliaa.alrashidi@qu.edu.sa; 3 Department of Pharmaceutics, College of Pharmacy, Prince Sattam Bin Abdulaziz University, P.O. Box 173, Al-Kharj 11942, Saudi Arabiaalbarraknourah3@gmail.com (N.A.A.);; 4 Bioenergy Research Unit, Department of Biophysics, Institute for Research and Medical Consultations, Imam Abdulrahman Bin Faisal University, P.O. Box 1982, Dammam 31441, Saudi Arabia; 5 Department of Chemistry, Faculty of Applied Sciences, Taiz University, Taiz 6803, Yemen; mn.alansi2016@gmail.com

**Keywords:** sunlight-assisted synthesis, *Cucurbita pepo* peel, steady-state photoluminescence, excitation–emission matrix fluorescence, antibacterial nanomaterials

## Abstract

Sunlight-assisted plant-mediated synthesis of silver nanoparticles (AgNPs) is well established, yet the complementary use of fluorescence spectroscopy to examine the optical response of complex phytochemical-containing media remains of interest. Here, *Cucurbita pepo* peel extract (CPE) was used for rapid AgNP formation under natural sunlight using single-step (A-Sun) and dropwise (B-Sun) addition strategies. The characteristic localized surface plasmon resonance (LSPR) band developed near 445–450 nm within 5 min, while corresponding dark controls showed negligible nanoparticle formation over the same period. Both dispersions retained their characteristic LSPR response during six months of optical monitoring. Steady-state photoluminescence (PL) measurements at excitation wavelengths of 300–350 nm and excitation–emission matrix (EEM) fluorescence mapping revealed lower recorded fluorescence intensities in the order CPE > A-Sun > B-Sun, with the principal emission region remaining broadly preserved near 435–440 nm. These observations provide complementary fluorescence fingerprints of the investigated preparations; however, inner-filter effects and light scattering may contribute to the measured attenuation, preventing its exclusive assignment to molecular quenching. FTIR revealed changes in oxygen-containing functional-group vibrations, while TEM showed predominantly spherical to quasi-spherical AgNPs, with mean particle diameters of 11.88 ± 3.60 nm for A-Sun and 9.77 ± 3.43 nm for B-Sun. The resulting dispersions also exhibited antibacterial activity against the six investigated bacterial strains. Overall, integrating PL and EEM with conventional characterization provides additional information on fluorescence changes accompanying sunlight-assisted AgNP formation without directly establishing the individual phytochemical reducing species, electron-transfer steps, or molecular surface-capping mechanisms.

## 1. Introduction

Silver nanoparticles (AgNPs) are among the most extensively investigated metallic nanomaterials because their size-dependent optical, surface, catalytic, and biological properties enable applications spanning antimicrobial technologies, sensing, environmental remediation, food-related systems, and biomedical materials [1,2,3]. Their characteristic localized surface plasmon resonance (LSPR) provides a sensitive optical signature of nanoparticle formation and surrounding dielectric environment, while their antimicrobial performance has been associated with multiple pathways involving Ag^+^ release, membrane interactions, oxidative stress, and disruption of essential cellular processes [1,2]. These multifunctional characteristics continue to motivate the development of synthesis strategies that combine controllable nanoparticle formation with reduced chemical and energy requirements.

Conventional physical and chemical routes for AgNP production can require strong reducing agents, stabilizers, elevated temperatures, or energy-intensive processing. Plant-mediated synthesis offers an attractive alternative because naturally occurring biomolecules can participate simultaneously in Ag^+^ reduction and nanoparticle stabilization under comparatively mild conditions [2,3,4]. Plant extracts contain complex mixtures of phenolic compounds, flavonoids, carbohydrates, proteins, terpenoids, organic acids, and other metabolites whose hydroxyl and oxygen-containing functionalities can participate in electron transfer, metal-ion coordination, and surface capping [2,4]. However, this compositional complexity also creates a fundamental challenge: although nanoparticle formation can readily be demonstrated, identifying how the phytochemical environment evolves during reduction and stabilization remains considerably more difficult.

Agricultural by-products provide an especially attractive phytochemical resource because their utilization couples nanomaterial synthesis with waste valorization. Pumpkin peels are commonly reported to contain bioactive constituents and antioxidant activity [5,6]. In particular, *Cucurbita pepo* peel has been reported to contain phenolic compounds and to exhibit antioxidant activity [6]. Converting this underutilized biomass into functional nanomaterials therefore offers a route toward integrating green nanotechnology with circular-material concepts while reducing dependence on dedicated synthetic reducing and capping reagents.

Despite extensive progress in plant-mediated AgNP synthesis, much of the literature continues to establish nanoparticle formation and properties primarily through UV–Vis spectroscopy, FTIR, XRD, electron microscopy, and related structural or surface-characterization techniques [2,3,4]. These methods are indispensable, but they provide limited direct information on how the complex fluorescent phytochemical population responds optically during AgNP formation.

Fluorescence spectroscopy offers a complementary opportunity because changes in emission intensity and excitation–emission behavior can reflect alterations in the fluorescent environment, although optical attenuation and scattering may also influence the recorded signals. Excitation–emission matrix (EEM) spectroscopy is particularly valuable for complex fluorescent mixtures because it maps the fluorescence response across both excitation and emission wavelengths rather than at only one selected excitation wavelength [7]. Although fluorescence and EEM approaches have been applied to silver-containing systems, their systematic integration with steady-state PL, FTIR, microscopy, and stability measurements to interrogate fluorescence changes in phytochemical-containing media accompanying plant-mediated AgNP formation remains comparatively underexplored.

This gap is particularly relevant for light-assisted green synthesis. Natural sunlight offers a sustainable photonic energy source for materials synthesis and photochemical transformations, reducing reliance on artificial irradiation and external energy inputs [8,9]. In phytogenic silver nanoparticle synthesis, solar irradiation can accelerate phytochemical-mediated Ag^+^ reduction, and several studies have demonstrated sunlight-driven AgNP formation using biological reducing systems [10,11,12]. Reported reaction times vary substantially with the biological source and reaction conditions, ranging from only a few minutes to tens of minutes or longer. Importantly, rapid nanoparticle appearance alone does not explain how the fluorescence characteristics of the complex phytochemical environment change during nanoparticle formation or whether the principal fluorescence signatures are retained after AgNP formation. Thus, advancing sunlight-assisted green synthesis requires moving beyond “whether nanoparticles form” toward interrogating the accompanying fluorescence responses of phytochemical-containing media.

Accordingly, the present work investigates rapid AgNP formation from *Cucurbita pepo* peel extract (CPE) under direct natural sunlight, with particular emphasis on fluorescence-assisted characterization of changes in the phytochemical-containing medium accompanying nanoparticle formation. Single-step and controlled dropwise addition of CPE to the AgNO_3_ solution were compared to examine differences in the recorded fluorescence response and nanoparticle morphology between the two preparation routes. Rather than relying solely on conventional confirmation of AgNP formation, steady-state photoluminescence (PL) at selected excitation wavelengths, two-dimensional EEM fluorescence mapping, and three-dimensional fluorescence profiling were employed as complementary spectroscopic probes of changes in the recorded fluorescence fingerprint of the investigated dispersions.

These observations were integrated with UV–Vis spectroscopy, FTIR, TEM, EDS elemental mapping, and six-month optical stability measurements to provide complementary insight into the optical, chemical, and morphological characteristics accompanying sunlight-driven AgNP formation. The antibacterial activity against representative Gram-positive and Gram-negative bacteria was subsequently evaluated to assess the antibacterial response of the resulting AgNPs. The distinctive contribution of this study lies in integrating steady-state PL and multidimensional EEM fluorescence profiling with conventional spectroscopic and microscopic characterization to interrogate changes in the overall fluorescence response of the complex phytochemical-containing medium accompanying rapid sunlight-driven AgNP formation, without assigning these changes to a specific molecular reduction or surface-stabilization mechanism.

## 2. Experimental Method

### 2.1. Materials

Fresh *Cucurbita pepo* (pumpkin) fruits were purchased from a local market in Al-Kharj, Saudi Arabia. The peels were used as the source of phytochemicals for silver nanoparticle synthesis. Silver nitrate (AgNO_3_, ≥99.9%, analytical grade) was purchased from Sigma-Aldrich (St. Louis, MO, USA). Absolute ethanol was supplied by Merck (Darmstadt, Germany). Deionized (DI) water was used throughout the study for solution preparation and washing procedures. Nutrient agar (Oxoid, Basingstoke, UK), nutrient broth (M002, HiMedia Laboratories Pvt. Ltd., Mumbai, India), and Mueller–Hinton agar (HiMedia Laboratories Pvt. Ltd., Mumbai, India) were used without further purification.

### 2.2. Preparation of Cucurbita pepo Peel Extract

Fresh pumpkin peels were washed thoroughly with tap water followed by deionized water to remove surface contaminants. The cleaned peels were cut into small pieces and dried under ambient laboratory conditions until constant weight was achieved. The dried material was ground into a fine powder using a laboratory grinder.

To prepare the phytochemical extract, 7.5 g of peel powder was dispersed in a mixed solvent consisting of 200 mL deionized water and 100 mL absolute ethanol. The suspension was heated at 60 °C under continuous magnetic stirring for 3 h to facilitate extraction of the bioactive compounds. After cooling naturally to room temperature, the extract was filtered through Whatman No. 1 filter paper. The filtrate was collected in an amber glass bottle and stored at 4 °C. The extract was freshly prepared and used within 48 h to minimize possible phytochemical degradation.

### 2.3. Sunlight-Assisted Green Synthesis of Silver Nanoparticles

Silver nanoparticles (AgNPs) were synthesized using the prepared *Cucurbita pepo* peel extract as both the reducing and stabilizing agent without the addition of any external reducing or capping chemicals.

A freshly prepared 1.0 mM aqueous AgNO_3_ solution was used for all synthesis experiments. For each experiment, 37.5 mL of the AgNO_3_ solution was transferred into a clean glass beaker and stirred magnetically at room temperature. Subsequently, 12.5 mL of the peel extract was introduced using two different addition strategies: 

Sample A (single-step addition): the entire extract volume was rapidly added to the AgNO_3_ solution under continuous stirring.

Sample B (dropwise addition): the same extract volume was added slowly dropwise while maintaining identical stirring conditions. The total reaction volume for each synthesis was 50 mL.

Immediately after mixing, the reaction mixtures were exposed to direct natural sunlight for 5 min. All sunlight-assisted synthesis experiments were conducted in Al-Kharj, Saudi Arabia, during October, between 9:00 and 10:00 AM under clear-sky conditions. The average solar irradiance during the experiments was approximately 90 mW cm^−2^, measured using the solar-radiation sensor of a Vantage Pro2 Plus ISS solar power meter (Model 6328, Davis Instruments, Hayward, CA, USA).

Natural sunlight, containing ultraviolet (UV), visible (Vis), and near-infrared (NIR) components, served as the sole irradiation source for nanoparticle synthesis. The reaction vessels were positioned to receive comparable solar exposure, and all experiments were performed under similar environmental conditions to minimize variations in sunlight exposure and reaction conditions. The reduction of Ag^+^ ions was visually indicated by a gradual color change from pale yellow to dark brown. Nanoparticle formation was initially monitored by this color transformation and subsequently evaluated using UV–Vis spectroscopy.

**Figure 1:**
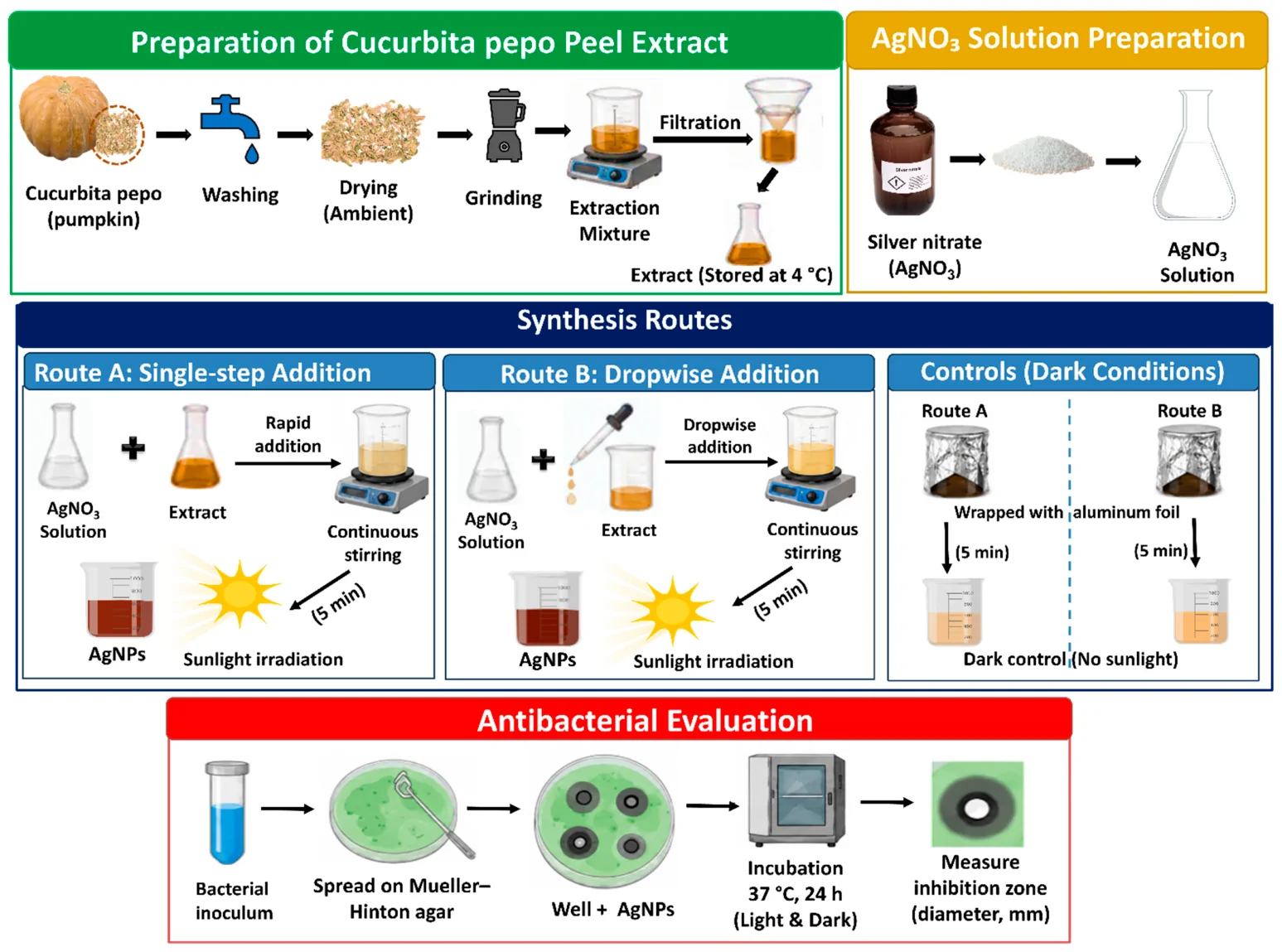
Schematic overview of *Cucurbita pepo* peel extract (CPE) preparation, AgNO_3_ solution preparation, and sunlight-assisted AgNP synthesis using two preparation strategies: single-step addition (sample A) and dropwise addition (sample B) of CPE to AgNO_3_. The workflow also illustrates the corresponding dark-control conditions and subsequent antibacterial evaluation.

To evaluate the contribution of sunlight, identical reaction mixtures were prepared under dark conditions by completely wrapping the reaction vessels with aluminum foil while maintaining all other experimental parameters unchanged. The dark-treated reaction mixtures showed negligible evidence of nanoparticle formation within the same 5 min reaction period based on the corresponding UV–Vis response. Therefore, only the sunlight-prepared samples (A-Sun and B-Sun) were selected for subsequent characterization and antibacterial evaluation. Following synthesis, the AgNP dispersions were stored in tightly capped amber glass bottles at approximately 4 °C, protected from light. Their optical stability was monitored by UV–Vis spectroscopy after 1 h, 3 months, and 6 months of storage. The overall experimental workflow, including CPE preparation, AgNO_3_ solution preparation, the two sunlight-assisted synthesis routes, dark controls, and antibacterial evaluation, is schematically illustrated in Figure 1.

### 2.4. Control Experiments

To clarify the individual roles of natural sunlight and phytochemicals in nanoparticle formation, control experiments were conducted using AgNO_3_ solution alone and *Cucurbita pepo* peel extract (CPE) alone under both sunlight and dark conditions. Each control solution was exposed to identical experimental conditions for 5 min, matching those employed during AgNP synthesis. The samples were subsequently analyzed by UV–Vis spectroscopy to compare the optical responses of the individual components with those of the complete reaction mixtures and to assess the contribution of sunlight and CPE to AgNP formation within the investigated reaction period. These controls were used to assess the optical evidence of nanoparticle formation within the investigated 5 min period and were not intended to establish the individual molecular steps involved in sunlight-assisted Ag^+^ reduction.

### 2.5. Characterization

The synthesized AgNPs and the crude CPE were characterized using complementary analytical techniques to investigate their optical, structural, chemical, and elemental properties. UV–Vis absorption spectra were recorded using a Jasco V-730 UV–Vis spectrophotometer (Jasco Corporation, Tokyo, Japan) over the wavelength range of 300–800 nm to monitor the localized surface plasmon resonance (LSPR) and evaluate the long-term optical stability of the AgNP suspensions. Photoluminescence (PL) spectroscopy was performed using a Jasco FP-8250 spectrofluorometer (Jasco Corporation, Tokyo, Japan). Steady-state PL emission spectra at excitation wavelengths of 310 and 340 nm were selected for comparative presentation. Excitation–emission matrix (EEM) fluorescence measurements were acquired over an excitation range of 300–350 nm and an emission range of 370–590 nm, and the resulting datasets were visualized as two-dimensional EEM maps and three-dimensional fluorescence profiles. Identical acquisition conditions were applied to CPE, AgNPs A-Sun, and AgNPs B-Sun to facilitate comparison of the recorded fluorescence responses. The fluorescence intensities were interpreted as measured optical responses without applying a validated correction for primary or secondary inner-filter effects or nanoparticle scattering.

Fourier-transform infrared (FTIR) spectra were collected using a Nicolet iS5 FTIR spectrometer (Thermo Fisher Scientific, Waltham, MA, USA) equipped with an iD5 ATR accessory over the spectral range of 4000–400 cm^−1^. The morphology, particle size, and particle distribution of the synthesized AgNPs were examined using a Talos F200i transmission electron microscope (Thermo Fisher Scientific, Eindhoven, The Netherlands). TEM image analysis was performed using Velox software (version 3.22.2, Thermo Fisher Scientific). The elemental distribution of the selected AgNP sample was investigated by energy-dispersive X-ray spectroscopy (EDS) elemental mapping using the EDAX APEX system (EDAX, LLC, Pleasanton, CA, USA).

### 2.6. Antibacterial Evaluation

The antibacterial activity of the biosynthesized AgNP suspensions was evaluated using the agar well-diffusion method against three Gram-positive bacteria (*Staphylococcus aureus*, *Bacillus subtilis*, and *Enterococcus faecalis*) and three Gram-negative bacteria (*Klebsiella pneumoniae*, *Pseudomonas aeruginosa*, and *Escherichia coli*). Fresh bacterial cultures were adjusted to 0.5 McFarland (approximately 1.5 × 10^8^ CFU mL^−1^) and uniformly inoculated onto Mueller–Hinton agar plates. Wells of 7 mm diameter were prepared for CPE, AgNPs A-Sun, and AgNPs B-Sun. For each treatment, 30 μL of freshly prepared suspension was added directly without centrifugation, washing, or dilution. CPE served as the extract-only control. Based on the synthesis formulation (37.5 mL of 1.0 mM AgNO_3_ in a final reaction volume of 50 mL), the nominal silver-equivalent concentration was 0.75 mM, corresponding to approximately 2.43 µg of theoretical silver input per 30 µL aliquot. This calculated value does not represent an experimentally determined AgNP mass, residual Ag^+^ concentration, or bioavailable silver dose.

The plates were incubated at 37 °C for 24 h under artificial LED illumination or dark conditions. A commercially available 40 W LED lamp was used as the illumination source, positioned at an estimated distance of approximately 35 cm from the agar surface. Inhibition-zone diameters were measured using a SCAN 500 automated inhibition-zone reader (Interscience, Saint-Nom-la-Bretèche, France). The recorded diameters were evaluated relative to the 7 mm well diameter to distinguish inhibition extending beyond the well from the well opening itself. One documented measurement was available for each treatment, bacterial strain, and incubation condition; therefore, the results are presented descriptively without statistical significance testing. A positive antibiotic control, MIC/MBC measurements, and matched AgNO_3_ and solvent-vehicle controls were not included in the available experimental dataset. Accordingly, the antibacterial findings are interpreted as preliminary screening observations rather than definitive measurements of antibacterial potency.

## 3. Results and Discussion

### 3.1. Rapid Sunlight-Assisted Formation of Silver Nanoparticles: Visual and UV–Vis Evidence

**Figure 2:**
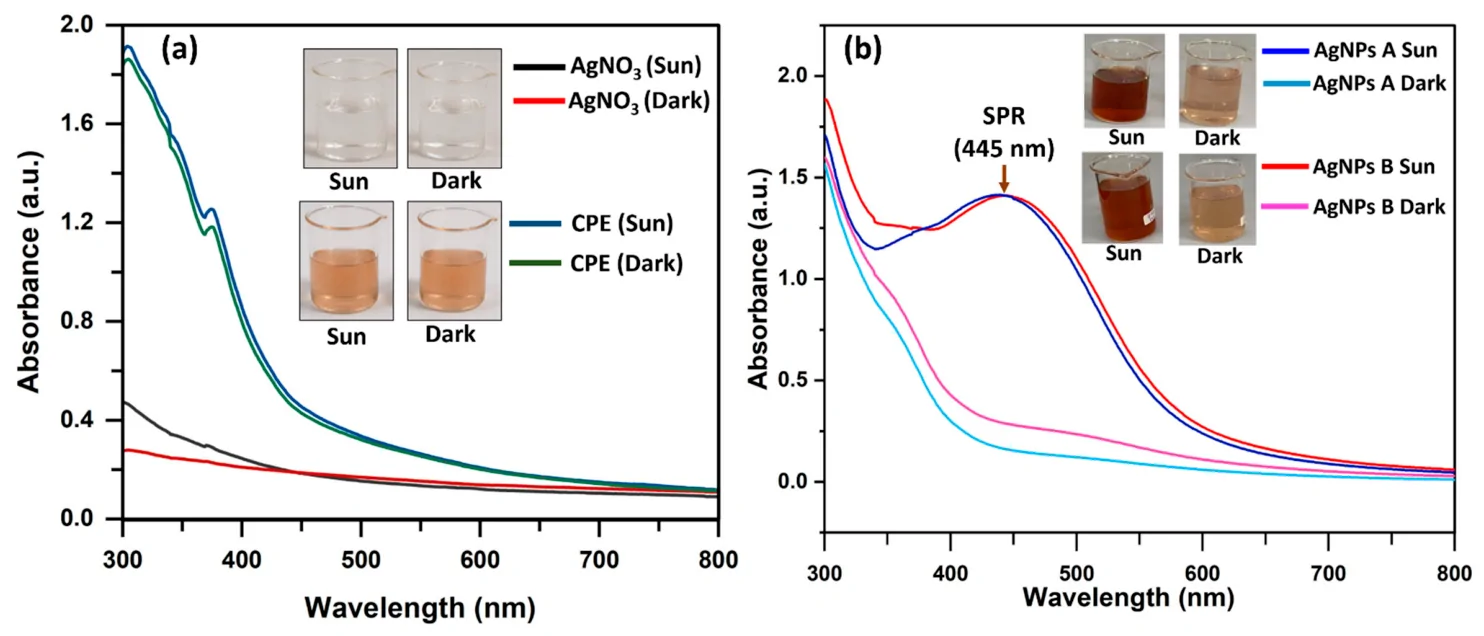
Visual appearance and UV–Vis spectra of the precursor solutions and AgNPs synthesized under sunlight and dark conditions. (**a**) AgNO_3_ solution and CPE. (**b**) AgNPs prepared by single-step addition (Sample A) and dropwise addition (Sample B). The appearance of an LSPR band at ~445 nm in the sunlight-treated mixtures, together with its absence in the plotted dark controls, is consistent with sunlight-associated AgNP formation under the stated conditions; it does not identify the molecular reduction steps.

The early stage of AgNP formation was investigated by visual observation and UV–Vis spectroscopy to evaluate the contributions of natural sunlight and CPE to AgNP formation. Figure 2 compares the precursor controls and reaction mixtures after 5 min under sunlight and dark conditions. As shown in Figure 2a, AgNO_3_ remained visually colorless under both conditions and showed no characteristic AgNP-associated plasmonic band in the visible region. CPE likewise exhibited comparable coloration and UV–Vis profiles after sunlight and dark treatment, with strong UV absorption associated with its complex phytochemical composition. Thus, 5 min of sunlight exposure alone did not produce a substantial change in the recorded UV–Vis response of either precursor.

A markedly different response occurred when CPE and AgNO_3_ were combined (Figure 2b). Both A-Sun and B-Sun developed a reddish-brown coloration within only 5 min of natural sunlight exposure, whereas the corresponding dark samples remained comparatively pale. Such color development is commonly associated with the plasmonic response accompanying plant-mediated AgNP formation [13,14]. Consistently, both sunlight-treated samples exhibited a pronounced absorption band centered at approximately 445 nm, while no comparable band appeared in the dark-treated mixtures. This band is consistent with the localized surface plasmon resonance (LSPR) characteristic of AgNPs. Plant-mediated AgNPs commonly exhibit LSPR bands within approximately 400–500 nm, with the exact position influenced by particle size, morphology, aggregation, and the surrounding dielectric environment [15,16,17,18].

The controls therefore demonstrate that the strong plasmonic response was observed only when CPE, AgNO_3_, and sunlight were combined within the investigated 5 min reaction period. The results are consistent with sunlight-assisted AgNP formation in the presence of phytochemical constituents, which may participate in Ag+ reduction and nanoparticle stabilization [1,19]. However, these optical controls do not independently resolve the elementary photochemical steps involved in the reduction process. Both A-Sun and B-Sun exhibited LSPR maxima at approximately 445 nm, with broadly comparable spectral profiles (Figure 2b), indicating similar characteristic plasmonic responses for the two synthesis routes under the investigated conditions.

### 3.2. Morphological and Structural Characterization of AgNPs

**Figure 3:**
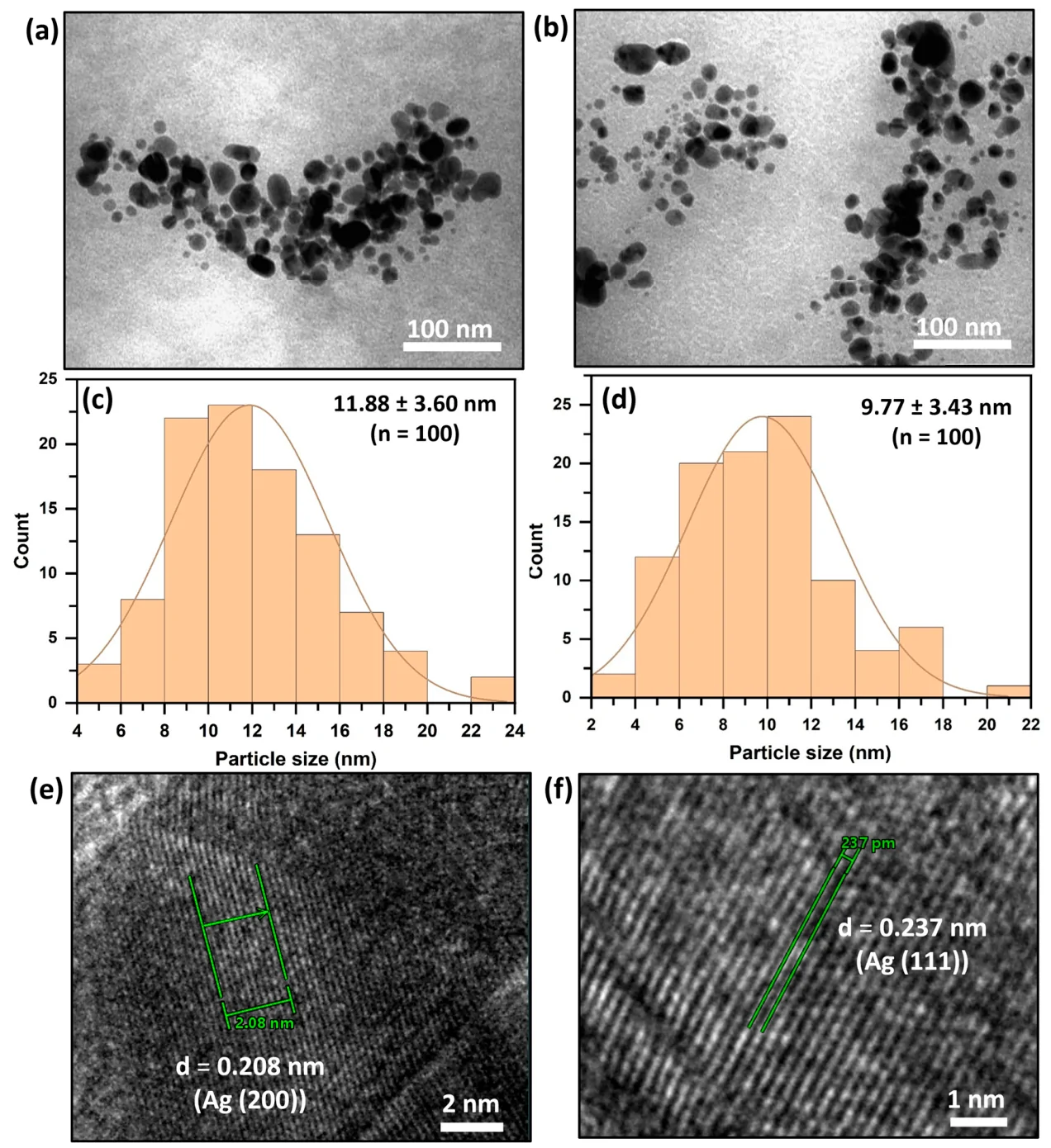
TEM and HRTEM characterization of sunlight-synthesized AgNPs. (**a**,**b**) TEM images of A-Sun (single-step addition) and B-Sun (dropwise addition), respectively. (**c**,**d**) Particle-size distributions showing mean diameters of 11.88 ± 3.60 and 9.77 ± 3.43 nm, respectively (mean ± standard deviation; *n* = 100 particles per sample). (**e**,**f**) HRTEM images of A-Sun and B-Sun showing lattice spacings of 0.208 and 0.237 nm, respectively. These local spacings are compatible with the (200) and (111) planes of fcc Ag, respectively, but do not establish bulk phase composition or phase purity.

Transmission electron microscopy (TEM) was employed to examine the morphology and particle-size characteristics of AgNPs synthesized through the two sunlight-assisted synthesis strategies. As shown in Figure 3a,b, both A-Sun and B-Sun contain predominantly spherical to quasi-spherical nanoparticles, with some particles appearing in clustered arrangements [18,20,21]. Particle-size analysis was performed on TEM micrographs by measuring 100 identifiable nanoparticles for each sample. The corresponding particle-size distributions are presented in Figure 3c,d. The particle counts describe the analyzed particle populations and do not represent independent synthesis batches.

The mean particle diameters were 11.88 ± 3.60 nm for A-Sun and 9.77 ± 3.43 nm for B-Sun (mean ± standard deviation; *n* = 100 particles per sample). The measured diameter ranges were 4.98–23.44 nm and 3.56–20.69 nm, respectively. These measurements indicate that B-Sun exhibited an approximately 17.8% smaller mean particle diameter than A-Sun within the analyzed particle populations. Although the absolute standard deviation was slightly lower for B-Sun (3.43 nm) than for A-Sun (3.60 nm), the coefficients of variation were 35.1% and 30.3%, respectively. Thus, the measured particle populations support a smaller mean diameter for B-Sun, but do not establish a narrower relative particle-size distribution or improved colloidal stability. The reported standard deviations describe particle-size variability within the analyzed TEM populations rather than batch-to-batch synthesis reproducibility.

High-resolution transmission electron microscopy (HRTEM) was further employed to examine local crystalline ordering (Figure 3e,f). For A-Sun, the HRTEM image (Figure 3e) revealed lattice fringes with an estimated interplanar spacing of 0.208 nm, calculated from a measured distance of 2.08 nm spanning ten consecutive lattice intervals. This value is close to the reference spacing of approximately 0.204 nm for the (200) planes of face-centered cubic (fcc) metallic Ag [22]. For B-Sun, the HRTEM image (Figure 3f) showed a measured lattice spacing of 0.237 nm, closely matching the reference spacing of approximately 0.236 nm for Ag(111) [23]. These measured lattice spacings are consistent with local crystalline domains of fcc metallic Ag in both samples. Nevertheless, HRTEM examines selected nanoscale regions and cannot independently establish the overall phase composition, demonstrate phase purity, or exclude minor silver-containing phases.

The smaller mean particle diameter observed for B-Sun suggests an association between the addition strategy and the particle-size characteristics of the analyzed samples. Gradual addition may alter the local availability of CPE-derived reducing and stabilizing constituents within the AgNO_3_-containing reaction mixture, potentially influencing particle formation and growth.

**Figure 4:**
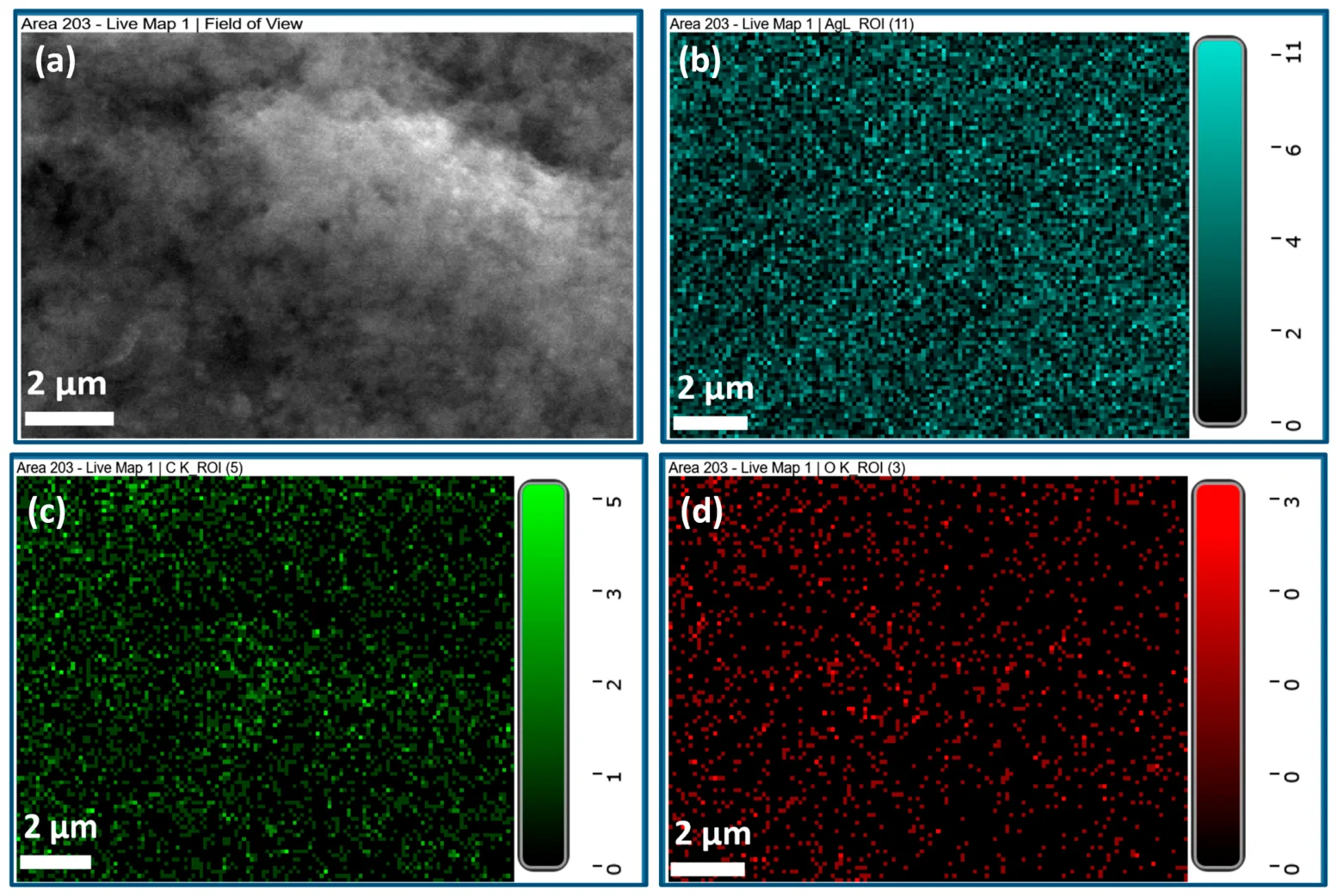
SEM–EDS characterization of B-Sun AgNPs: (**a**) SEM micrograph and (**b**–**d**) qualitative elemental maps assigned to Ag, C, and O, respectively. Scale bars: 2 µm. Original magnification: 19,600×.

Scanning electron microscopy coupled with energy-dispersive X-ray spectroscopy (SEM–EDS) was additionally used to examine the elemental distribution of a representative B-Sun specimen (Figure 4a–d). The SEM micrograph (Figure 4a) provides a micrometer-scale overview of the deposited AgNP-containing material, whereas the elemental maps (Figure 4b–d) illustrate the spatial distribution of Ag, C, and O, respectively. The detection of Ag supports the presence of silver-containing material within the analyzed region [21]. Carbon and oxygen signals were also detected, which may arise from CPE-derived organic constituents and/or other contributions associated with specimen preparation and analysis. Although these observations are compatible with the presence of organic material in the analyzed specimen, SEM–EDS does not establish the chemical identity, origin, or specific binding of these constituents to the AgNP surfaces. Overall, the combined TEM, HRTEM, particle-size analysis, and SEM–EDS observations support the formation of Ag-containing nanoparticles with differences in the measured particle-size distributions and local crystalline domains consistent with fcc metallic Ag, without establishing bulk phase purity.

### 3.3. Long-Term Optical Response of Sunlight-Synthesized AgNPs

**Figure 5:**
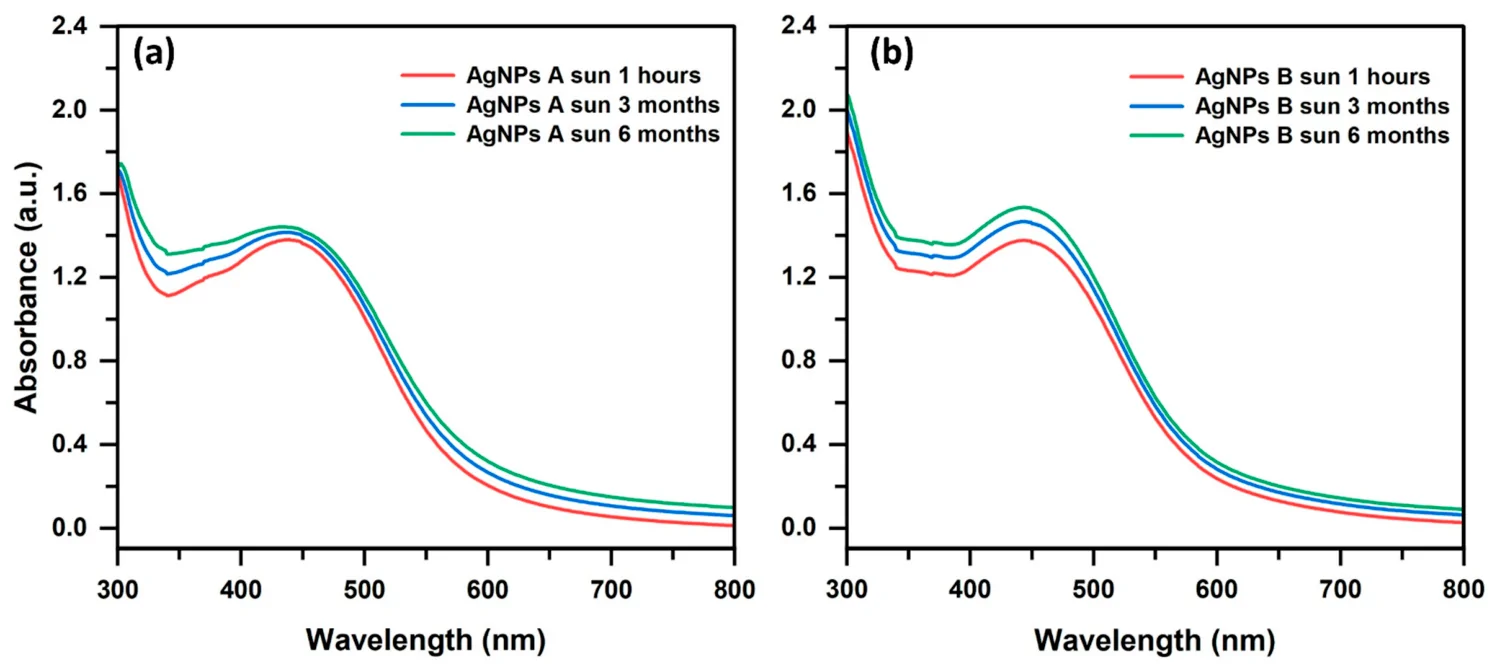
UV–Vis stability spectra of sunlight-synthesized AgNPs prepared by (**a**) single-step addition and (**b**) dropwise addition after 1 h, 3 months, and 6 months. Persistence of the LSPR band within ~440–450 nm demonstrates retention of the characteristic plasmonic response during the six-month monitoring period.

The long-term optical response of A-Sun and B-Sun was evaluated by monitoring their UV–Vis spectra after 1 h, 3 months, and 6 months of storage. As shown in Figure 5a,b, both samples retained a well-defined localized surface plasmon resonance (LSPR) band within the 440–450 nm region throughout the six-month monitoring period. Because the position and spectral profile of the AgNP LSPR are sensitive to changes in particle size, aggregation state, and local dielectric environment, their temporal evolution provides a useful spectroscopic indicator of changes in the nanoparticle dispersion [13,24].

Neither A-Sun (Figure 5a) nor B-Sun (Figure 5b) exhibited pronounced LSPR shifts or severe spectral broadening during the monitoring period. The absence of substantial changes in the LSPR position and profile indicates retention of the characteristic plasmonic response over six months [25]. Modest variations in absorbance intensity were observed; however, these were not assigned to a specific structural process because absorbance can also be influenced by particle concentration, dispersion state, and measurement conditions. The persistence of the LSPR band may reflect a stabilizing contribution from constituents of the CPE-containing reaction medium, although their specific role in the observed long-term optical response cannot be established from UV–Vis spectra alone. Plant metabolites can participate in metal-ion reduction and subsequently contribute to nanoparticle stabilization through surface association. Nevertheless, the present UV–Vis measurements demonstrate retention of the optical response rather than independently confirming unchanged particle size, aggregation state, or colloidal stability throughout storage.

### 3.4. FTIR Analysis of Phytochemical Functional Groups Associated with AgNP Formation

**Figure 6:**
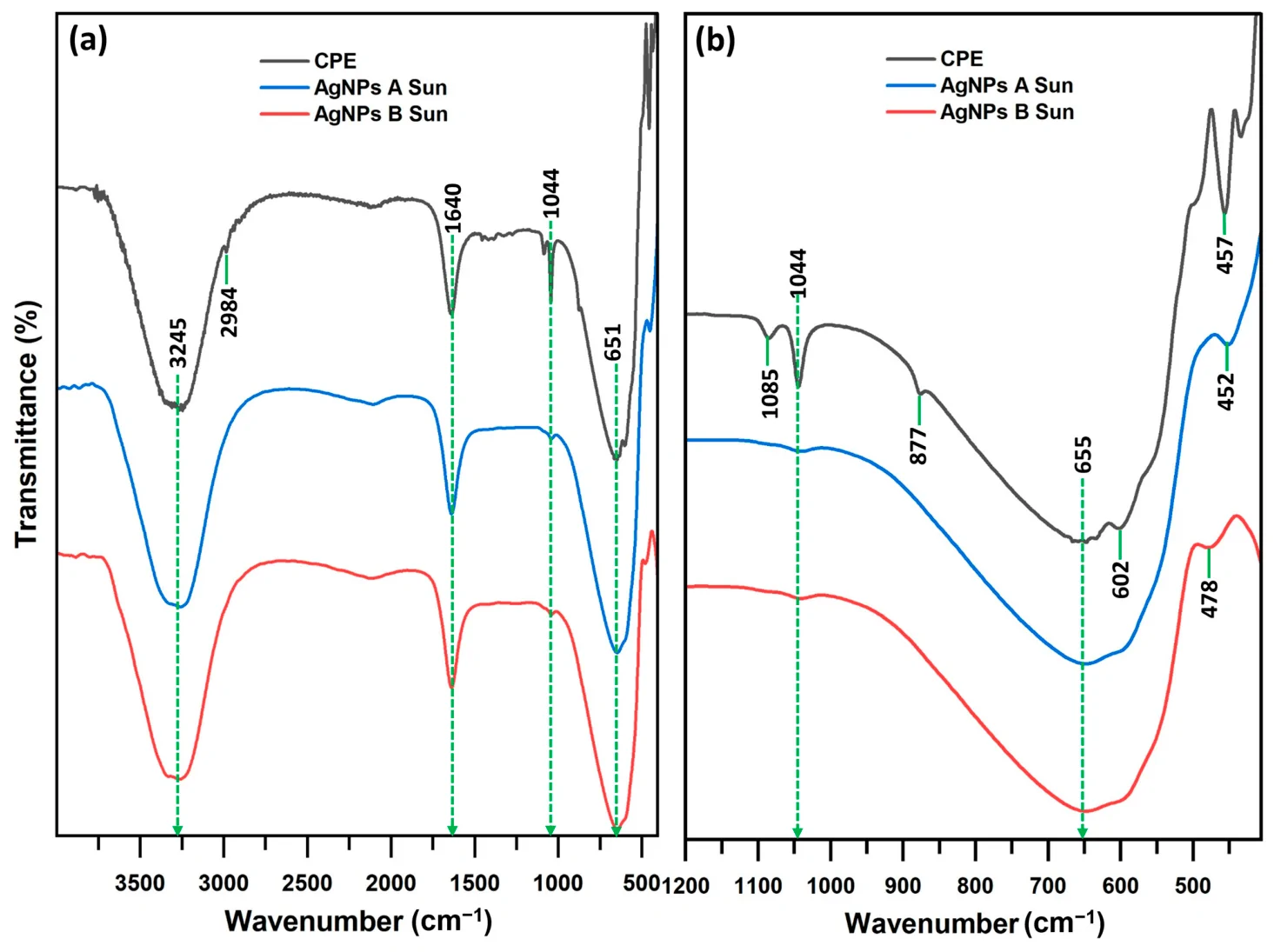
FTIR spectra of CPE and sunlight-synthesized AgNPs (Samples A and B): (**a**) full spectral range (4000–400 cm^−1^) and (**b**) enlarged fingerprint region (1200–400 cm^−1^), showing changes in O–H, C–O, and other phytochemical-associated vibrational features accompanying AgNP formation.

Fourier-transform infrared (FTIR) spectroscopy was used to examine changes in the vibrational features associated with functional groups in CPE and the AgNP-containing samples following sunlight-assisted AgNP formation (Figure 6). The CPE spectrum exhibits a broad band centered at approximately 3245 cm^−1^, mainly attributable to hydrogen-bonded O–H stretching of hydroxyl-containing phytochemicals. Broad O–H absorption within the ~3200–3400 cm^−1^ region is commonly reported for plant extracts and phytogenic AgNP systems [10,26]. Following AgNP formation, changes in the width and profile of this band indicate differences in the O–H-associated vibrational environment, consistent with the possible participation or surface association of hydroxyl-containing species.

The weak band at 2984 cm^−1^, assigned to aliphatic C–H stretching, becomes less evident after synthesis. In contrast, the prominent band near 1640 cm^−1^, which may include contributions from conjugated C=O/C=C vibrations and/or bound-water bending, remains at approximately the same position in A-Sun and B-Sun. Similar features in the 1620–1650 cm^−1^ region have been reported for phytochemical-mediated AgNP systems [10,27]. Its persistence suggests that the corresponding vibrational environment is broadly retained after nanoparticle formation.

More pronounced changes are observed in the C–O-rich spectral region. The CPE bands around ~1085 and ~1044 cm^−1^, attributable to C–O and C–O–C vibrations of oxygen-containing phytochemical functionalities, are strongly attenuated or no longer clearly resolved after AgNP formation. These changes suggest differences in the spectral contributions of C–O-containing functionalities between CPE and the AgNP-containing samples and are consistent with their possible involvement in nanoparticle formation or subsequent surface association. However, FTIR alone does not identify the individual phytochemical species responsible for these changes or distinguish unambiguously between reduction-related transformation and surface association.

Changes are also observed in the low-wavenumber region. The CPE feature near 655 cm^−1^ is markedly altered, while bands appear near 452 cm^−1^ for A-Sun and 478 cm^−1^ for B-Sun. Because this region may contain contributions from several metal–ligand and skeletal vibrations, these changes are conservatively attributed to alterations in the local bonding environment associated with AgNP formation rather than to a specific Ag–O vibration [12,28]. The FTIR changes, particularly those associated with O–H- and C–O-containing functionalities, are consistent with changes in the chemical environment of oxygen-containing constituents in the CPE-containing preparations following AgNP formation. These observations provide complementary vibrational information alongside the fluorescence measurements discussed in subsequent sections, without establishing a direct molecular relationship between the two sets of spectral changes.

### 3.5. Steady-State Photoluminescence of CPE and AgNPs

Photoluminescence (PL) spectroscopy was employed to investigate excitation-dependent changes in the fluorescence response of the CPE-containing preparations following sunlight-assisted AgNP formation. While UV–Vis spectroscopy establishes the characteristic plasmonic response associated with AgNP formation, steady-state PL provides complementary information on the emissive constituents of the reaction medium and their overall optical response. Phenolic and flavonoid compounds have been reported to participate in plant-mediated Ag^+^ reduction, while fluorescence spectroscopy is sensitive to changes in the local environment of fluorescent organic constituents [29]. The fluorescence of CPE may originate from a heterogeneous population of fluorophores, potentially including phenolic, flavonoid, and other conjugated phytochemical species. Comparable blue-region fluorescence associated with plant-derived fluorophores has been reported in phytogenic AgNP systems [12].

**Figure 7:**
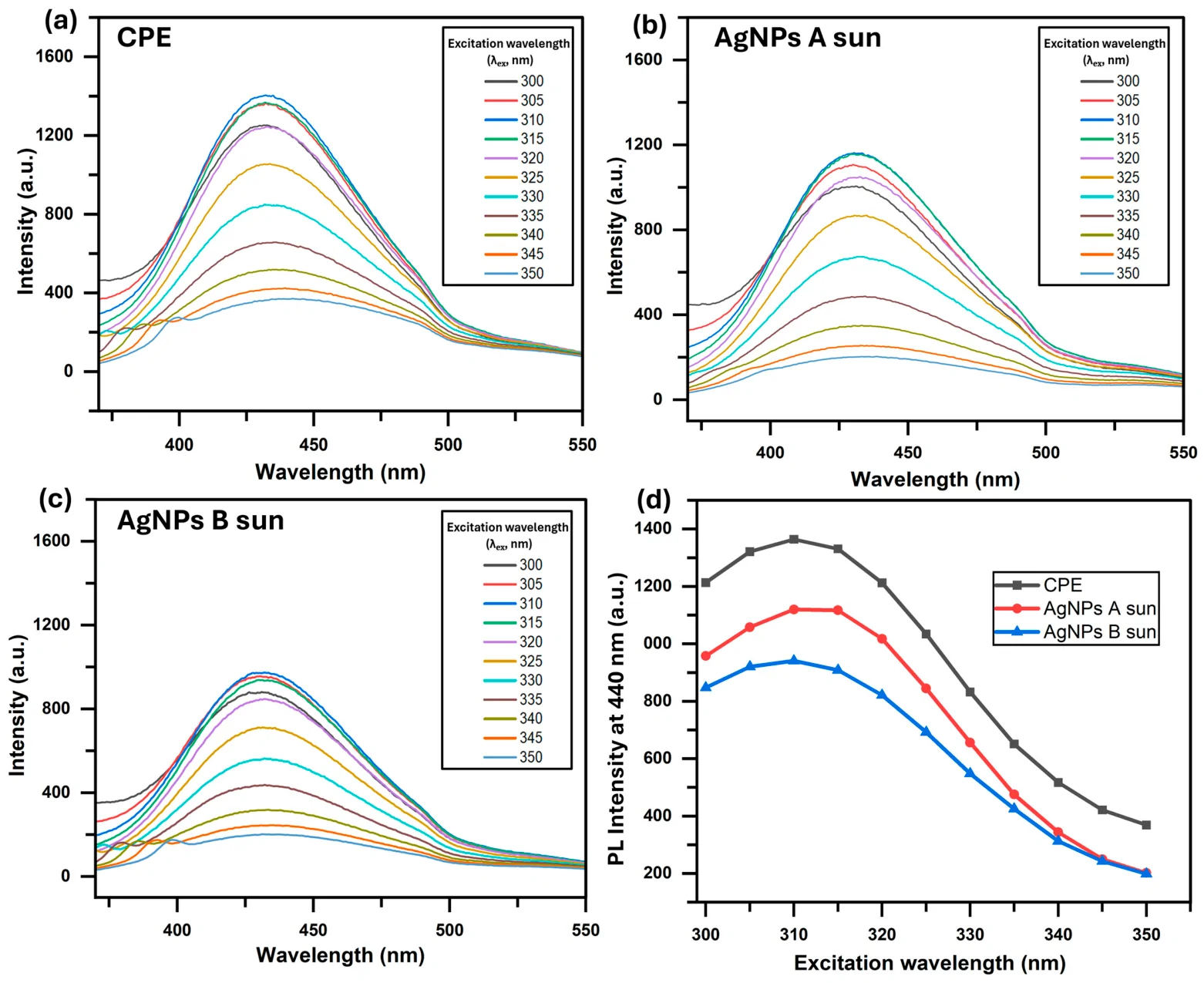
Excitation-dependent steady-state photoluminescence (PL) spectra of (**a**) *Cucurbita pepo* peel extract (CPE), (**b**) sunlight-synthesized AgNPs prepared by single-step addition (A-Sun), and (**c**) sunlight-synthesized AgNPs prepared by dropwise addition (B-Sun), recorded at excitation wavelengths (λex) ranging from 300 to 350 nm in 5 nm increments. (**d**) Comparison of PL intensities at a fixed emission wavelength of 440 nm as a function of excitation wavelength for CPE, A-Sun, and B-Sun.

To obtain a broader excitation-dependent fluorescence profile rather than relying on a single excitation wavelength, PL emission spectra were recorded at 11 excitation wavelengths from 300 to 350 nm in 5 nm increments. Figure 7a–c presents the corresponding spectra for CPE, A-Sun, and B-Sun, respectively. All three preparations exhibit broad emission bands centered approximately within the 430–440 nm region, with the dominant emission region remaining broadly preserved after AgNP formation. The broad spectral features are compatible with overlapping contributions from fluorescent constituents in the complex CPE-containing media, although individual fluorophores cannot be resolved or identified from these measurements alone. In contrast to the broadly similar emission-band positions, the recorded fluorescence intensities show pronounced excitation dependence and clear differences among the three preparations.

Figure 7d summarizes the variation in the recorded PL intensity at a fixed emission wavelength of 440 nm as a function of excitation wavelength for the investigated preparations. The highest intensities within the measured excitation range occur at 310 nm, followed by a progressive decrease toward 350 nm. Across all 11 investigated excitation wavelengths, the recorded intensities follow the sequence CPE > A-Sun > B-Sun. Notably, B-Sun exhibits lower recorded fluorescence intensity than A-Sun throughout this range, despite the broadly comparable LSPR maxima of the two samples. Thus, steady-state PL measurements at different excitation wavelengths provide an additional optical distinction between the two addition strategies that is not apparent from comparison of their LSPR peak positions alone.

An essential consideration in interpreting these results is that the observed intensity decrease cannot be assigned exclusively to molecular fluorescence quenching. The AgNP-containing dispersions exhibit appreciable optical absorbance and may scatter excitation and emitted light, potentially affecting the measured PL intensities. The recorded steady-state PL spectra were not corrected for primary or secondary inner-filter effects. To assess the potential contribution of these effects, absorbance values at a representative excitation wavelength (310 nm) and near the principal emission maximum (440 nm) were obtained from the UV–Vis data for the investigated preparations. At 310 nm, the absorbance values were 1.875, 1.485, and 1.721 for CPE, A-Sun, and B-Sun, respectively, whereas the corresponding values at 440 nm were 0.497, 1.414, and 1.410, respectively. These values indicate substantial differences in optical absorbance among the preparations at both wavelengths. Consequently, primary inner-filter effects arising from attenuation of excitation light and secondary inner-filter effects associated with reabsorption of emitted light, together with possible scattering contributions, may influence the measured fluorescence-intensity differences.

Because no validated inner-filter correction, dilution-dependent fluorescence measurements, or fluorescence lifetime analysis was performed, the respective contributions of optical attenuation and molecular quenching cannot be quantitatively separated. Accordingly, the sequence CPE > A-Sun > B-Sun is interpreted as an observed difference in steady-state fluorescence intensity under the investigated experimental conditions, rather than evidence of progressively stronger molecular quenching or more efficient electron transfer. The present PL data also cannot independently establish the extent of phytochemical adsorption, molecular surface coverage, fluorescence quantum yield changes, or the specific identity of surface-associated compounds.

Nevertheless, the observed excitation-dependent differences remain useful as comparative optical fingerprints of the investigated preparations. Although A-Sun and B-Sun show similar characteristic LSPR responses, their PL profiles differ consistently in recorded intensity across the excitation range. These differences may reflect a combination of changes in the fluorescent constituents or their local environments and differences in the optical properties of the resulting AgNP-containing dispersions. Phenolic acids and flavonoids, including chlorogenic acid and quercetin derivatives, have been reported to participate in Ag^+^ reduction in plant-mediated systems [30]. However, the present measurements do not establish the presence or specific involvement of these individual compounds in the investigated CPE system. Similarly, the differences in particle-size distributions observed by TEM (Section 3.2) do not independently demonstrate a causal relationship between nanoparticle diameter and the measured fluorescence attenuation.

The principal analytical contribution of the present PL investigation is the systematic comparison of 11 excitation-dependent emission spectra for the original CPE and both sunlight-assisted AgNP preparations, rather than the observation of fluorescence attenuation alone. Although fluorescence responses of phytogenic AgNP systems have been reported previously [20], the present multi-excitation analysis demonstrates that the recorded CPE > A-Sun > B-Sun intensity sequence is maintained across the investigated excitation domain and provides a complementary means of distinguishing the two precursor-addition strategies despite their similar LSPR maxima. This excitation-dependent approach offers a more comprehensive descriptive fluorescence fingerprint than a single-excitation measurement, while explicitly recognizing the contributions that inner-filter effects and scattering may make to the recorded intensities. The subsequent EEM analysis extends this comparison into the excitation–emission domain, enabling the spatial distribution of fluorescence features to be examined alongside the excitation-dependent emission spectra. Together, these complementary fluorescence measurements strengthen the optical characterization of sunlight-assisted AgNP formation without claiming direct identification of the molecular reduction, quenching, or surface-capping mechanisms.

### 3.6. Excitation–Emission Matrix Fluorescence Analysis

**Figure 8:**
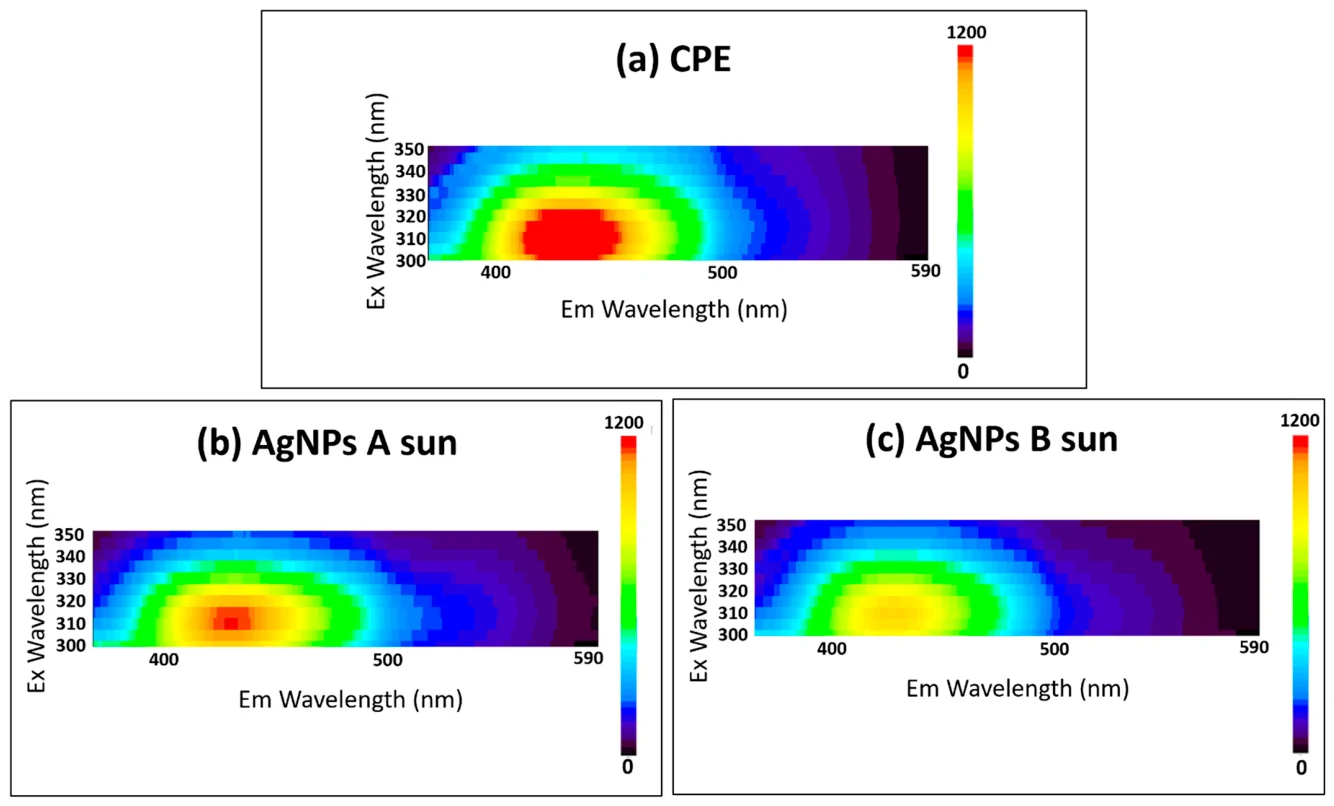
Two-dimensional EEM fluorescence maps of (**a**) *Cucurbita pepo* peel extract (CPE), (**b**) single-step (A-Sun), and (**c**) dropwise (B-Sun) sunlight-synthesized AgNPs. All maps share a common intensity scale (0–1200 a.u.), showing a broadly preserved fluorescence domain with decreasing intensity (CPE > A-Sun > B-Sun).

Excitation–emission matrix (EEM) fluorescence spectroscopy was employed to obtain a multidimensional fluorescence fingerprint of CPE and to examine changes in the recorded fluorescence response of the CPE-containing preparations following AgNP formation. Complementing the excitation-dependent steady-state PL measurements discussed in Section 3.5, EEM analysis represents fluorescence intensity across both excitation and emission dimensions, providing a broader visualization of the fluorescence characteristics of complex mixtures [31]. The EEM maps of CPE, A-Sun, and B-Sun are presented in Figure 8a–c, respectively. All three maps are displayed using a common fluorescence-intensity scale of 0–1200 a.u., allowing the recorded intensity distributions to be compared visually. CPE (Figure 8a) exhibits the strongest fluorescence response, with a broad high-intensity domain extending approximately across Ex = 300–340 nm and Em = 400–500 nm. The most intense region is located approximately within Ex = 305–320 nm and Em = 420–450 nm, as estimated from the displayed map. The broad fluorescence envelope may reflect overlapping contributions from several fluorescent constituents in the crude plant extract, including potentially phenolic, flavonoid, and other conjugated compounds [12]. However, the observed fluorescence domain does not permit the identification of individual phytochemical species.

Following AgNP formation, the displayed EEM maps reveal a progressive reduction in the recorded fluorescence intensity. In A-Sun (Figure 8b), the high-intensity red–orange region becomes less prominent than in CPE, whereas B-Sun (Figure 8c) exhibits a further decrease, with its principal fluorescence domain represented predominantly by yellow–green and cyan colors. Under the common displayed intensity scale, these observations are consistent with the intensity sequence CPE > A-Sun > B-Sun previously identified from the excitation-dependent PL measurements (Figure 7). An additional observation from the EEM maps is the broad preservation of the principal fluorescence domain across the three preparations. Although the apparent extent of the high-intensity region decreases from CPE to A-Sun and B-Sun, the dominant fluorescence response remains concentrated within a broadly similar excitation–emission region, particularly around Ex = 300–325 nm and Em = 420–450 nm. No clearly separated new dominant fluorescence domain is apparent within the spectral window investigated. These observations suggest that the principal differences among the displayed fluorescence fingerprints involve the magnitude and distribution of recorded intensity rather than the emergence of a distinct new emission feature. Nevertheless, the absence of a resolved new domain does not exclude changes in individual fluorophores whose emission bands may overlap within the broad fluorescence envelope.

The apparent contraction of the high-intensity region should also be interpreted cautiously. Because the displayed colors represent fluorescence-intensity intervals, a reduction in the area occupied by high-intensity colors does not independently demonstrate a decrease in fluorophore concentration, selective consumption of phytochemicals, or chemical transformation of particular molecular species. Furthermore, the maps provide a qualitative comparison of the measured intensity distributions rather than a quantitative assessment of integrated fluorescence intensity or individual peak coordinates, which would require analysis of the original numerical EEM data. As discussed in Section 3.5, the measured fluorescence attenuation cannot be attributed exclusively to molecular quenching. The appreciable and non-equivalent optical absorbance of CPE, A-Sun, and B-Sun at relevant excitation and emission wavelengths indicates that primary and secondary inner-filter effects may influence the recorded fluorescence intensities. In addition, light scattering by the AgNP-containing dispersions may contribute to the observed differences. The EEM data were not corrected for these effects, and their relative contributions cannot be quantitatively separated using the available measurements. Consequently, the EEM maps are interpreted as comparative, uncorrected fluorescence fingerprints rather than direct evidence of molecular quenching, electron transfer, or specific phytochemical–AgNP binding interactions.

Despite these limitations, EEM analysis provides complementary insight into the fluorescence characteristics of the two synthesis routes. Although A-Sun and B-Sun exhibit similar LSPR peak positions, their EEM maps reveal distinguishable intensity distributions, with B-Sun showing a weaker principal fluorescence domain. By combining multi-excitation PL (Figure 7) with EEM mapping (Figure 8), the present study provides a more comprehensive fluorescence fingerprint, capturing both excitation-dependent intensity variations and their distribution across the excitation–emission domain. However, these differences do not independently establish specific phytochemical–AgNP interactions or surface-stabilization mechanisms. Together with the TEM and FTIR results (Section 3.2 and Section 3.4), this integrated analysis strengthens the optical characterization of the sunlight-synthesized AgNPs without assigning unsupported molecular mechanisms.

### 3.7. Proposed Sunlight-Assisted AgNP Formation Pathway

**Figure 9:**
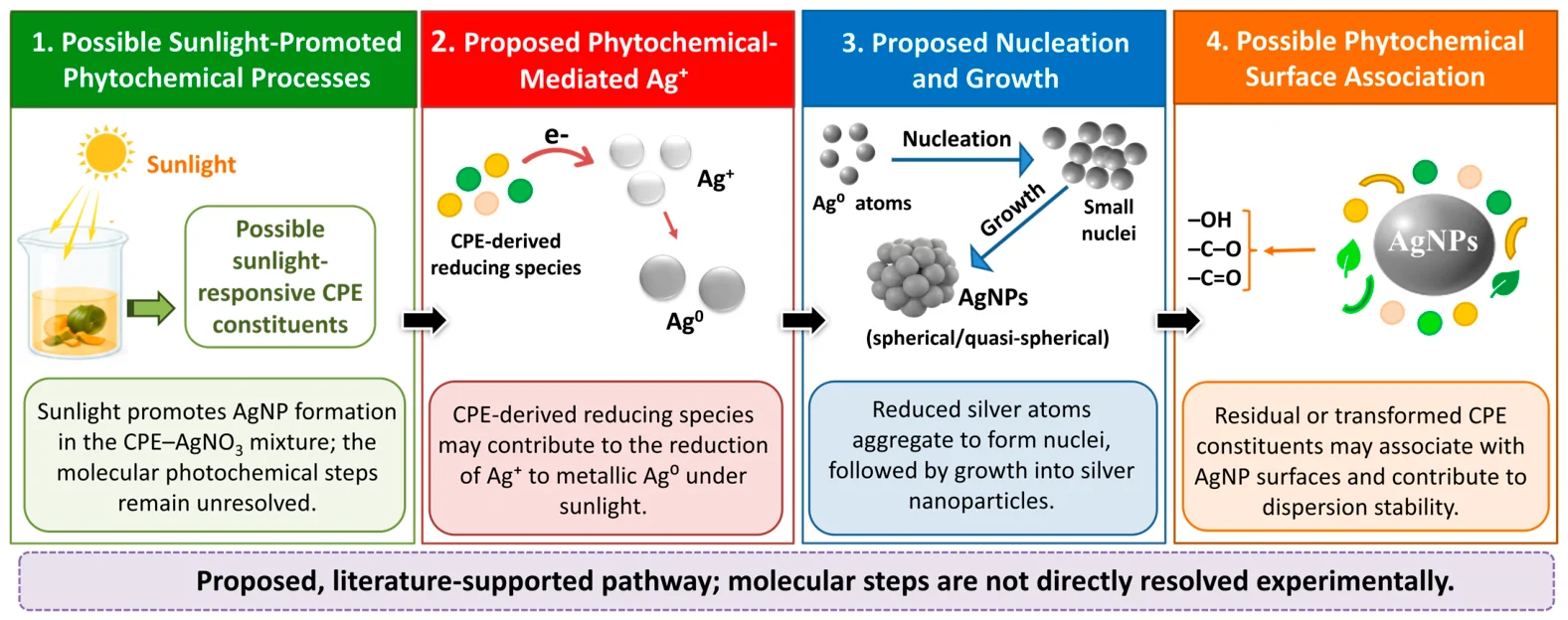
Proposed pathway for sunlight-assisted AgNP formation using CPE, illustrating phytochemical-mediated Ag^+^ reduction, nanoparticle nucleation and growth, and surface stabilization.

Based on the light/dark control experiments and the combined UV–Vis, TEM, FTIR, PL/EEM, and SEM–EDS observations, a literature-supported pathway is proposed to explain the sunlight-assisted formation of AgNPs using CPE (Figure 9). Under the investigated 5 min reaction conditions, the CPE–AgNO_3_ mixture developed a characteristic LSPR band near 445 nm upon sunlight exposure, whereas the corresponding dark control showed no comparable plasmonic response. This observation supports a strong promoting role of sunlight in AgNP formation over the examined reaction period, without identifying the underlying photochemical steps. Similar sunlight-assisted phytochemical reduction processes have been proposed in previous studies [10,28,32].

The proposed pathway involves four interconnected stages: (i) possible sunlight-promoted changes in redox-active CPE constituents, (ii) phytochemical-mediated reduction of Ag^+^ to Ag^0^, (iii) nucleation and growth of AgNPs, and (iv) possible association of residual or transformed phytochemicals with the nanoparticle surfaces. Sunlight may facilitate the reducing activity of CPE-derived constituents, while the resulting Ag^0^ species may participate in nanoparticle nucleation and growth [16,32,33,34,35]. However, photoexcited phytochemical intermediates, individual electron donors, and nucleation kinetics were not directly identified in this study. The observed LSPR response, predominantly spherical to quasi-spherical TEM morphologies, and local HRTEM lattice spacings consistent with fcc metallic Ag support the formation of AgNPs, but do not establish the proposed sequence of elementary reactions.

FTIR revealed changes in O–H- and C–O-associated vibrational features, while SEM–EDS mapping detected Ag, C, and O within the examined B-Sun specimen. These observations are compatible with the presence of CPE-derived organic constituents in the nanoparticle-containing material, although they do not establish the identity or binding configuration of specific surface-associated molecules. The characteristic LSPR band remained detectable after six months of storage, demonstrating retention of the plasmonic response rather than independently proving a particular molecular stabilization mechanism [13,36]. The PL and EEM measurements provide complementary fluorescence fingerprints of the preparations, with recorded intensities following CPE > A-Sun > B-Sun. However, because inner-filter effects and scattering may contribute to this attenuation, the fluorescence results are not used as direct evidence of phytochemical photoactivation, electron transfer, or surface capping. Overall, Figure 9 summarizes a plausible pathway consistent with the experimental observations and previous literature, while distinguishing the demonstrated sunlight-enhanced AgNP formation from the proposed molecular-level processes.

### 3.8. Antibacterial Activity of Sunlight-Synthesized AgNPs

**Figure 10:**
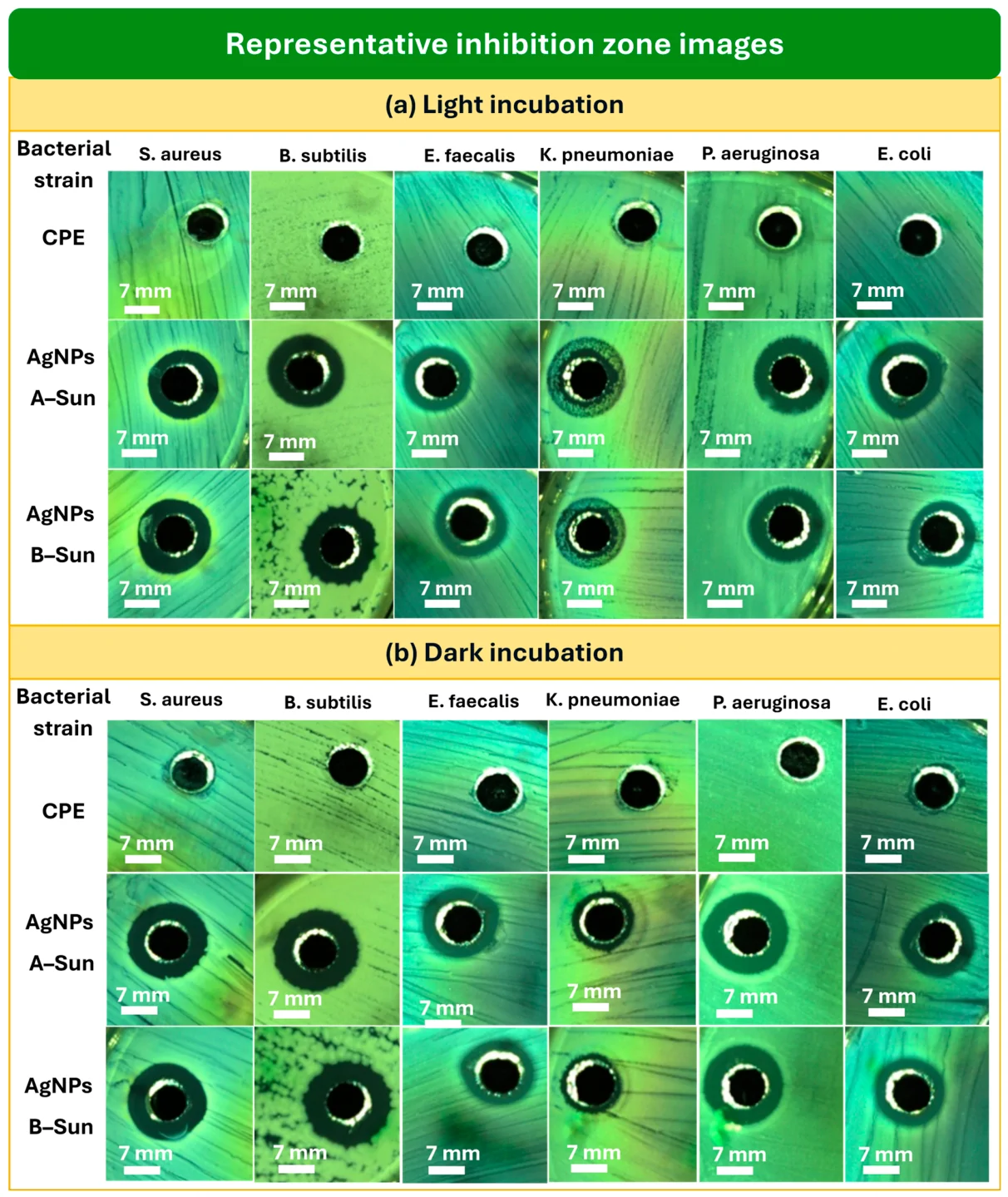
Representative inhibition zones of CPE and sunlight-synthesized AgNPs (A-Sun and B-Sun) against six bacterial strains under (**a**) light and (**b**) dark incubation conditions. Scale bars: 7 mm.

**Figure 11:**
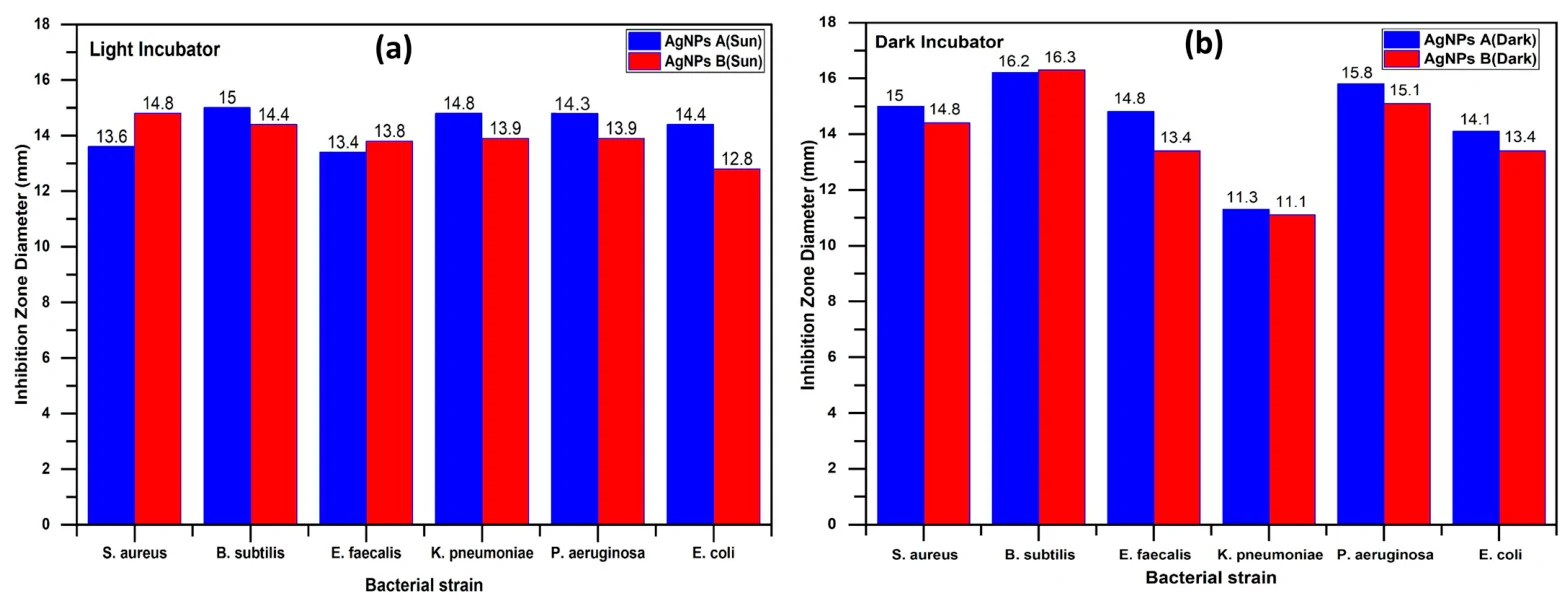
Comparison of inhibition zone diameters of AgNP Samples A and B against different bacterial strains under (**a**) light and (**b**) dark incubation.

The antibacterial activity of the sunlight-synthesized silver nanoparticles was evaluated against three Gram-positive bacteria (*Staphylococcus aureus*, *Bacillus subtilis*, and *Enterococcus faecalis*) and three Gram-negative bacteria (*Klebsiella pneumoniae*, *Pseudomonas aeruginosa*, and *Escherichia coli*) using the agar well diffusion method under light and dark incubation conditions. Representative photographs of the inhibition zones are presented in Figure 10, while the corresponding quantitative comparison is summarized in Figure 11. The complete numerical values are listed in Table 1.

The crude CPE exhibited no detectable inhibition zone under the tested conditions, whereas the AgNP-containing samples generated distinct inhibition zones against all six bacterial strains (Figure 10). These results demonstrate an antibacterial response of the AgNP-containing samples under the conditions of the agar-diffusion assay, whereas CPE alone showed no detectable activity. Broad-spectrum antibacterial responses of plant-mediated AgNPs against both Gram-positive and Gram-negative bacteria have been widely reported and may be influenced by nanoparticle physicochemical properties, surface-associated constituents, Ag^+^ release, and diffusion behavior within the agar medium [35].

Under light incubation (Figure 11a), inhibition-zone diameters ranged from 12.8 to 15.0 mm. A-Sun showed numerically larger zones against *B. subtilis*, *K. pneumoniae*, *P. aeruginosa*, and *E. coli*, whereas B-Sun showed numerically larger zones against *S. aureus* and *E. faecalis*. The numerical differences between A-Sun and B-Sun were generally modest and did not show a consistent synthesis-route-dependent pattern across the six bacterial strains. Under dark incubation (Figure 11b), inhibition-zone diameters ranged from 11.1 to 16.3 mm. A-Sun showed numerically larger zones against *S. aureus*, *E. faecalis*, *K. pneumoniae*, *P. aeruginosa*, and *E. coli*, whereas B-Sun showed a slightly larger zone against *B. subtilis*.

Both AgNP-containing samples produced measurable inhibition zones under both incubation conditions. No consistent light-dependent enhancement was apparent across all tested strains from the agar-diffusion measurements. Accordingly, the present results are interpreted as comparative antibacterial screening rather than as evidence that illumination enhances or is required for antibacterial action. Similarly, neither synthesis route showed a consistently larger inhibition zone across all bacterial strains. Because independent replicate measurements were not available, the observed numerical differences were not subjected to statistical significance testing.

Because agar well diffusion is influenced by both antibacterial effects and the transport of the tested material through the agar matrix, inhibition-zone diameter should not be interpreted as a direct quantitative measure of intrinsic antibacterial potency. Furthermore, the present assay does not independently resolve the contributions of intact nanoparticles, released Ag^+^, or CPE-derived surface-associated constituents to the observed antibacterial response. Determination of MIC/MBC values and comparison with an appropriate positive antibiotic control would provide additional quantitative information on antibacterial potency. Previous studies have attributed AgNP antibacterial activity to multiple possible pathways, including interactions with bacterial envelopes, Ag^+^ release, oxidative stress, and subsequent disruption of cellular components [36]. These mechanisms were not directly measured in the present study and are therefore discussed only as literature-supported explanations that may contribute to the observed antibacterial response.

## 4. Conclusions

This study demonstrates the feasibility of rapid, sunlight-assisted AgNP synthesis using *Cucurbita pepo* peel extract as an agricultural-waste-derived resource. The development of a characteristic LSPR band within 5 min, together with the corresponding light/dark controls, establishes the promoting role of natural sunlight under the investigated reaction conditions. Both single-step (A-Sun) and dropwise (B-Sun) addition strategies produced predominantly spherical to quasi-spherical nanoparticles, with a smaller mean TEM-measured particle diameter for B-Sun (9.77 ± 3.43 nm) than for A-Sun (11.88 ± 3.60 nm). Both preparations retained their characteristic plasmonic response during six months of optical monitoring, although this observation alone does not establish long-term colloidal or structural stability.

The principal analytical contribution of this work is the integration of multi-excitation steady-state PL and EEM fluorescence mapping to characterize changes accompanying AgNP formation in a complex plant-extract medium. Despite broadly similar LSPR characteristics, the two synthesis routes exhibited distinguishable recorded fluorescence responses, following the sequence CPE > A-Sun > B-Sun, while the principal emission region remained broadly preserved. These findings demonstrate the complementary value of fluorescence fingerprinting beyond conventional LSPR analysis, without attributing the measured attenuation exclusively to molecular quenching. Inner-filter effects and scattering may contribute to the observed differences, while the combined fluorescence and FTIR observations do not identify individual reducing species or establish specific photoactivation, electron-transfer, or surface-capping mechanisms.

Both AgNP preparations exhibited inhibition zones against the six investigated bacterial strains under light and dark incubation, whereas CPE alone showed no detectable inhibition. The antibacterial findings support activity under the tested agar-diffusion conditions but do not establish intrinsic potency, a consistent advantage of either synthesis route, or a general light-dependent enhancement. Overall, the study establishes a rapid sunlight-assisted preparation approach and demonstrates the usefulness of complementary fluorescence analysis for comparing phytogenic AgNP preparations. Future investigations incorporating absorbance-corrected or time-resolved fluorescence, molecular-level phytochemical identification, independent colloidal-stability measurements, and quantitative antibacterial assays would help resolve the underlying mechanisms and assess the broader applicability of the resulting nanomaterials.

## Figures and Tables

**Table 1:** Inhibition zone diameters (mm) of CPE and AgNPs against the tested bacterial strains under light and dark incubation.

Bacterial Strain	Light Incubation			Dark Incubation		
	CPE	AgNPs A–Sun	AgNPs B–Sun	CPE	AgNPs A–Sun	AgNPs B–Sun
*S. aureus*	0	13.6	14.8	0	15.0	14.8
*B. subtilis*	0	15	14.4	0	16.2	16.3
*E. faecalis*	0	13.4	13.8	0	14.8	13.4
*K. pneumoniae*	0	14.8	13.9	0	11.3	11.1
*P. aeruginosa*	0	14.3	13.9	0	15.8	15.1
*E. coli*	0	14.4	12.8	0	14.1	13.4

Note: A value of 0 indicates no detectable inhibition zone beyond the 7 mm well diameter.

## Data Availability

The data presented in this study are available from the corresponding author upon reasonable request.
